# The Ripple Effect: A Train-the-Trainer Model to Exponentially Increase Organizational Faculty Development

**DOI:** 10.15694/mep.2020.000158.1

**Published:** 2020-08-05

**Authors:** Jessica Servey, Jessica Bunin, Thomas McFate, K. Christopher McMains, Rechell Rodriguez, Joshua Hartzell

**Affiliations:** 1 Uniformed Services University of the Health Sciences; 2 Uniformed Services University of the Health Sciences; Walter Reed National Military Medical Center; 3 Uniformed Services University of the Health Sciences; San Antonio Uniformed Services Health Education Consortium; South Texas Veterans Health Care System; 4 Uniformed Services University of the Health Sciences; VA San Diego Healthcare System

**Keywords:** Faculty development, train-the-trainer, residency, deliberate practice

## Abstract

This article was migrated. The article was marked as recommended.

Introduction:

Faculty development is a key component of undergraduate and graduate medical education and is required for accreditation. Many institutions face the challenges of training large numbers of faculty at multiple locations on a recurring basis. In order to meet the faculty development demands of our organization, we implemented a train-the trainer model of faculty development.

Methods:

A train-the-trainer program was created using deliberate practice as the theoretical framework. The primary goals of the program were to enhance content knowledge and develop facilitation skills of the participants (called faculty trainers). Two separate cohorts received 40 hours of in-person training consisting of attending the faculty development workshops as a learner, providing feedback to course faculty, facilitating and participating in journal club sessions on relevant content, and practicing facilitation and receiving feedback on the workshops. Cohorts 1 and 2 were trained on how to deliver 6 and 7 workshops, respectively. An additional 16 hours of training and further feedback occurred when faculty trainers delivered the workshops at outside institutions.

Results:

Twenty-nine faculty trainers from 15 specialties and subspecialties were trained, including 18 in the first cohort (January 2018) and 14 in the second cohort (February 2019) with 3 who participated in both cohorts. From January 2018 to January 2020, faculty trainers delivered 298 workshops to 3742 attendees at 25 locations. For the faculty trainers, 1477 evaluations were completed with 1031 (88.1%) rated as excellent, 141 (9.5%) rated as good, and 8 (0.5%) as average. There were no fair or poor ratings.

Discussion:

Our train-the-trainer program effectively developed a community of national faculty developers. Faculty trainer output was substantial and early evaluations of performance were positive. The model outlined in this paper serves as a potential sustainable model for other institutions desiring to train a cadre of faculty developers for their organization.

## Introduction

Requirements for faculty development in medical education continue to increase. These include foundational skills that most educators should be familiar with such as direct observation, creating a positive learning climate, and giving effective feedback. Additionally, there are certain skills that are necessary for program and institutional level leaders such as curriculum development, leadership, establishing programs of assessment, and developing wellness programs. Often, there are not enough faculty development opportunities to meet the needs of numerous educators with varied schedules and productivity demands of clinical practice. Furthermore, there are increasing requirements from accrediting bodies in medical and dental education for faculty development of all clinician educators ( Accreditation Council for Graduate Medical Education (ACGME), 2018), ( Liaison Committee on Medical Education (LCME), 2019), ( Commission on Dental Accreditation (CODA), 2019). The message is clear: no longer is expertise in a clinical area enough. All educators in the health professions would benefit from specific training in evidence-based educational principles, but institutions struggle to meet the needs of our educators.

Our organization is characterized by one medical school, and more than 200 graduate medical education (GME) programs conducting training in multiple outlying academic hospitals spread across the country. Additionally, faculty turnover rate is significantly higher than the national average ( Servey, McFate, and Reamy, 2018). As a result, historically, the organization has struggled to meet the ever-increasing demand for faculty development, especially for foundational educational topics such as feedback and learning climate. This problem led us to explore a train-the-trainer program for nationwide faculty development.

A train-the-trainer model has been used for faculty development by national organizations ( Osborn *et al.*, 2004) and universities ( Skeff *et al.*, 1992). Arguably the most well-known and longest standing train-the-trainer model is the Stanford Faculty Development Program (SFDP) developed by Stratos and Skeff ( Skeff *et al.*, 1992). The program has trained hundreds of facilitators, nationally and internationally over 30 years, who have subsequently trained thousands of faculty locally at their institutions. There have been other programs that have seen success with local programs ( Rosenbaum, Lenoch, and Ferguson, 2005). This model has also been used successfully outside of faculty development to increase medical knowledge and confidence. It has been used to educate physicians about end of life issues ( Stratos *et al.*, 2006) and pharmacists about pharmacogenomics ( Lee *et al*., 2012). Each of these programs train faculty to deliver content to an external department or institution, but after the training there is no formal continued relationship for administrative support or update of materials. We set out to create a train-the-trainer program with centralized administrative support, that could adapt and expand over time to meet the Faculty Development needs of a national organization.

## Methods

To meet the faculty development needs of one of the largest national health systems in the United States, we utilized a train-the-trainer model. The goal of the program was to develop a sustainable cadre of faculty developers who could deliver essential, foundational faculty development topics at 21 hospitals, where students of the Uniformed Services University of the Health Sciences are educated. This system has a national faculty of approximately 5000 individuals appointed to the School of Medicine. Four initial faculty developers, termed “master trainers” for the remainder of this manuscript, conducted two separate training events, covering distinct topics. Three of the master trainers were graduates of the Stanford Faculty Development Program (K. M., R. R., and J. H.). The learners in this program will be referred to as “faculty trainers” for the remainder of the manuscript. The first cohort consisted of 18 faculty trainers, who were selected via recommendation by the designated institutional official (DIO) of their academic hospital. The second cohort of 14 faculty trainers were also selected by the DIO of their hospital, with the additional criterion of having served five years as academic faculty.

The theoretical frameworks informing the instruction of the faculty trainers was deliberate practice ( Ericsson, 2008) and the experiential learning model based on Kolb’s learning styles ( Armstrong *et al.,* 2005). We created a program whereby the faculty trainers were given a well-defined goal, provided specific constructive feedback during training, and given multiple opportunities to iteratively practice, honing their facilitation skills with immediate, specific feedback. Our faculty trainers were motivated to improve, a requirement of deliberate practice theory. In our methodological approach to the program, we ensured the faculty trainers had various opportunities to self-reflect, engage in problem-solving, and develop plans for further personal improvements in facilitating faculty development.

The overarching goals of the training were to ensure content knowledge, develop facilitation skills of faculty trainers, and create a community of faculty developers. The two cohorts each received a 40-hour initial training course over a five-day period (See Supplementary File 1 for schedule). The initial five-day training consisted of attending the faculty development workshops as a learner, providing feedback to master trainers, facilitating and participating in journal club sessions on relevant content (See Supplementary File 2 for bibliography) and practicing facilitation of the actual workshops (See Table 1 for topics). The real-time practice presentations were videotaped and then critiqued by the individual faculty trainer with additional comments from peers and master trainers. In addition to the content and facilitations skills, several other skills were taught in the initial week, including: critiquing academic presentations, audio-visual technology use, self-assessment of teaching, and collaboration techniques. Initial training was followed by two days of additional training (totaling 16 hours) at an academic hospital other than the faculty trainers’ home hospital. Additional training days included presenting faculty development workshops in front of an unknown audience, providing peer feedback to master trainers (specifically querying facilitation techniques), continued training on content and technology challenges for presenters, and enhancing organizational understanding of their role. Although not planned deliberately, mentoring occurred as well throughout the process. In total, each faculty trainer received 56 hours of training and had multiple opportunities for direct observation and feedback of facilitations skills (deliberate practice).

**Table 1.** Faculty Development Train-the-Trainer Workshop Titles

Cohort 1	Cohort 2
Bedside Teaching	Active Learning
Feedback	Creating an Effective Poster
Large Group Teaching	Direct Observation
Learning Climate	Milestones and EPAS
Precepting	Role Modeling
Small Group Teaching	Supervision
	Writing Effective Narratives

To determine the content for the first cohort, the four master trainers identified foundational skills and concepts for clinician educators. Identified topics were then cross referenced to organizational level data to determine the most requested and facilitated workshops over the prior three years. For the second cohort, we decided to train the faculty trainers on additional topics instead of repeating the initial six. We aimed to increase the range and variety of content available to the faculty at each training hospital and to build on the original foundational topics. Within our organization, master trainers deliver additional annual faculty development on advanced topics throughout our network of training sites. With a broad range of foundational topics available through the faculty trainers, master trainers could focus on more advanced topics that broadened the educational skills of clinician educators throughout the system.

In the second year of the program, we began updating the first six workshops. In order to build community, faculty trainers updated workshops in teams of two. Each team subsequently trained their colleagues via a journal club and a presentation of the updated material. This served three distinct purposes: refresh new principles and literature, review changes in the teaching materials, and maintain standardization. Administrative assistants ensured removal of old materials as new ones were added in our learning management system.

Our program has instituted an annual review of each faculty trainer consistent with our model of deliberate practice. First, we created a set of expectations, then master trainers review feedback from the workshops with each trained faculty as well as ensuring program expectations are met. These conversations are springboards for goal setting for the next year.

## Results

A total of 29 faculty trainers were trained, 18 in the first and 14 in the second cohort (3 participated in both groups). Participants represented 12 of 21 (57.4%) of the organization’s academic hospitals. There were 15 specialties and subspecialties represented, including 2 dentists. The first cohort of faculty trainers began delivering workshops in January 2018 and the second cohort in February 2019. Between January 2018 and January 2020, faculty trainers delivered 298 workshops to 3742 attendees at 25 locations. Out of the original 29 faculty, one has moved into an enterprise-wide faculty development role, while two have decided not to continue teaching in the program. Ongoing efforts to expand the program continue. Sixteen of the faculty have chosen to participate in the expansion process, while ten have chosen to deliver only the material on which they were initially trained.

For all workshops, hard copy evaluations were distributed for hand-written feedback. Of the 1477 evaluations completed, 1031 (88.1%) rated the faculty trainers as “excellent,” 141 (9.5%) rated the faculty trainers as “good,” and 8 (0.5%) as average. No faculty trainer was rated as “fair” or “poor”. The remaining 1.8% were left blank at the Likert scale rating.

## Discussion

Our train-the-trainer program has been an effective model to develop a community of national faculty developers. The primary model for our course was the SFDP. This program is the most well-known and longitudinally rigorous train-the-trainer program, and fortunately, it aligns with our content. Although we used an approach similar to the SFDP ( Skeff *et al*., 1992), we had some distinct differences. In our program, there were two unique sets of workshops for the two cohorts. The course that we offered occurred over a week as opposed to the month scheduled by SFDP, which is a more feasible time frame for faculty to be away from their institutions for training. The number of workshops taught, and faculty attending have been significantly higher ( Skeff *et al*., 1992). The survey results of faculty performance imply that even with only a week of dedicated training our faculty trainers were able to deliver the material effectively. This suggests that others could develop a similar more condensed train-the-trainer program for faculty development at their institution.

**Figure 1.**
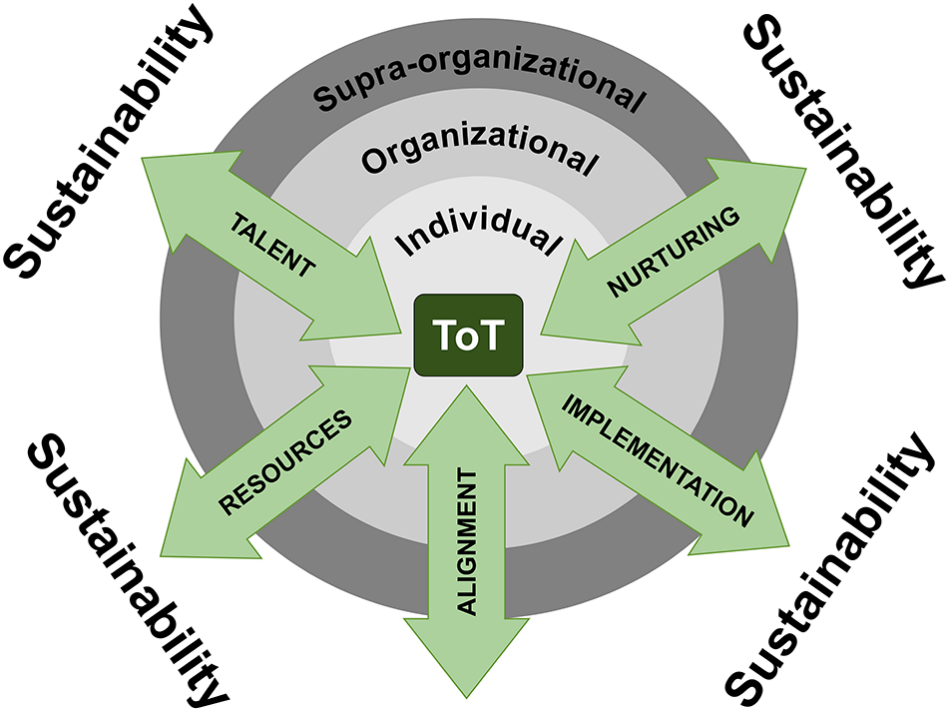
TRAIN framework.

The strengths of our program align with the TRAIN (talent, resources, alignment, implementation, nurturing) framework ( Mormina and Pinder, 2018) ( Figure 1), which we believe will aid in our strategic long-term sustainability plans. This framework has a tiered approach: individual, organizational and supra-organizational. To align with this framework, we identify the faculty trainers as the individuals, the Uniformed Services University Faculty Development Program and the Uniformed Services University national faculty as the organization, and the Military Health System (MHS) as the supra-organizational structure.

### Talent

On the individual level, our program was seeking enthusiastic, resilient individuals with academic aspirations, who were recognized as academic leaders in the local institutions. As such, we solicited input from DIOs at each institution to identify faculty trainer candidates who demonstrated a more strategic look at the program. After the first cohort, we identified additional requirements for faculty trainers to include increased experience levels within graduate medical education (greater than five years as faculty or a position of Associate Program Director or Program Director). In the future, our hope is a nomination package that aims to communicate the faculty nominee’s patience, receptiveness to feedback, and self-reflective skills. From the supra-organizational level, we looked at this as a talent management opportunity for the Military Health System. The hope was that selecting individuals for these roles would increase their sense of commitment to the MHS, provide them with leadership opportunities, and ultimately build the future GME and undergraduate medical education leaders for the MHS.

### Resources

Resourcing is an indispensable component to the success of this program. Protected time for training, preparation, and delivery of workshops is essential for the faculty trainers. While the individual trainers and the time that they dedicate are our greatest resource, the organization found that the key to sustainability is a centrally located administrative section. The central administration responsibilities consist of budgeting, maintenance of the learning management system, supervision of program evaluation needs, and direct support of the faculty trainers. The central budget supports educational material purchases and travel funding. The administrators continually update the learning management system as our organization grows and changes. They manage sign-in sheets and evaluations, provide information to the faculty trainers regarding the anticipated audience for their workshops, and manage continuing medical education and university faculty development certificate needs. An additional benefit from the central administrative support is that it necessitates fewer personnel. From the supra-organizational perspective, our hope is that with increasing recognition of the service provided by the faculty trainers, increased protected time and resources would be dedicated by the MHS. This, in turn, provides the MHS with the resource of well trained, committed, advanced medical educators.

### Alignment

Alignment is an essential principle of our program. The interests and motivations of the individual trainers must align with the organizational goals which have to align with the strategic objectives of the MHS. Enlisting the assistance of the Program Directors and DIOs in selection of faculty trainers was our first means of seeking this alignment. We wanted to ensure that the faculty trainers’ goals were consistent with the program, and that they were ultimately interested in pursuing an academic career. We recognized early that high levels of emotional intelligence and outstanding communication skills would be necessary for the individual faculty trainers to succeed with the training, at their local institutions, and as they traveled to outside hospitals. The DIOs were able to identify faculty trainers who were strong in these areas, but we also incorporated elements of these skills into the program.

From an organizational and supra-organizational perspective, we articulated the intention to create the train-the-trainer program to PDs and DIOs early in an attempt to ensure we were addressing the true needs of stakeholders. As the program grew, we maintained the lines of communication to ensure that we could adapt. For example, as the ACGME requirements grew to include specific areas for faculty development, we developed workshops in these areas to ensure national faculty could meet these requirements. For this reason, we chose to create seven new workshops for the second cohort of faculty trainers. This allowed faculty trainers to deliver a total of 13 workshops. To further meet the needs of our GME community, we created tracking programs so that we could report workshop attendance by national faculty back to the GME programs for accreditation purposes.

### Implementation

Early in the implementation phase, the focus was on the individual faculty trainers and optimizing the training they received. As described in our methods section, we instituted training based on the Kolb cycle ( Armstrong *et al*., 2005) as well as Ericsson’s deliberate practice model. In order to ensure material continued to meet the needs of the stakeholders and to ensure all material was current, we implemented new material and revision teams among the faculty trainers. This increased the commitment and community amongst the faculty trainers. This further displayed the program’s commitment to life-long learning and growth.

Institutional support for the implementation of the program was necessary in the form of protected time for faculty trainers. Without protected time, this would not have been possible and may be a barrier for some institutions. We continue to work within our organization and with the MHS (supra-organization) to ensure these faculty trainers are recognized for their efforts.

### Nurturing

Our program’s training did not conclude with the end of the initial intensive week. We continued to observe and give individual feedback. Prior to delivering the workshops individually, each faculty trainer traveled to an unfamiliar location to deliver workshops with the master trainer team. The additional time allowed more individual mentoring and relationship building. Annually, faculty trainers each have a feedback and coaching session with one of the master trainers after the evaluation forms for all workshops are reviewed. In addition to the coaching provided, mentoring relationships with the master trainers were also maintained to address issues of personal and professional development. We believe the ongoing support from the master trainers, and from peers, has been key to the sustainability of our program and to the continued growth of our faculty trainers. The continued relationships expand on other published train-the-trainer programs.

In addition to the support provided by the master trainers, the central administrators provide an additional route of ongoing nurturing and assistance. The administrators help with arranging all travel, managing schedules, and ensuring responsiveness for any issues that arise. The administrators also continue to help manage the online repository of talks and background materials.

Beyond the nurturing of the faculty trainers, the program itself requires nurturing. The program began branding its products and reporting its successes to stakeholders at various levels within the organization and supra-organization. Faculty trainers are encouraged to deliver educational workshops as teams at various society meetings and other conferences for their own professional development, as well as to increase recognition of the program. These talks provide further opportunities for deliberate practice.

### Challenges and Limitations

In our program, we experienced a few challenges which need to be acknowledged as another university or organization may choose to develop a similar program. First, there was some angst expressed by faculty trainers about using teaching materials developed by someone other than themselves. This is part of standardization and similar to the SFDC program ( Skeff *et al.*, 1992), yet the master trainers recognized this as a problem. We combatted this by allowing the faculty trainers to make a few defined changes to feel more authentic. Additionally, having teams of faculty trainers update the workshops enhanced comfort with the standardization process. A second challenge was that the faculty trainers did not report to the University leadership directly. Similar to other programs, the amount of faculty development each faculty trainer can deliver may be time limited by local hospital leadership. Another challenge is that the motivation varies among individual faculty trainers to deliver workshops, which workshops, and to what audience based on the confidence of that person. We attempted to optimize the challenge with transparent expectations for those nominating the faculty trainers, as well as clear (and later written) expectations to continue to be part of the program.

## Conclusions

A train-the-trainer model using the deliberate practice framework and the Kolb Cycle is an effective intervention to both magnify the number of foundational faculty development workshops available to our faculty, and to create highly skilled faculty developers. The model provides an example that other organizations could use to expand their faculty development teams.

## Take Home Messages

• A train-the trainer model can effectively increase faculty development in a standardized fashion for an organization or health system.
• Having a training plan based on the theory of deliberate practice can ensure confidence through practice and specific feedback.
• Using the TRAIN framework can help strategically consider individual, organizational and supraorganizational considerations for faculty development sustainability.

## Notes On Contributors

Jessica T. Servey, MD, MHPE is an Associate Professor of Family Medicine at the Uniformed Services University of the Health Sciences where she has served as the Associate Dean for Faculty Development for 6 years. Twitter - @jessicatsmom, ORCID: https://orcid.org/0000-0002-0092-7004

Jessica Bunin, MD is an Associate Professor of Medicine at the Uniformed Services University of the Health Sciences. She is currently the Assistant Dean for Faculty Development and the Program Director of the Critical Care Fellowship at Walter Reed National Military Medical System. Twitter - @jessica_bunin, ORCID: https://orcid.org/0000-0001-5978-8152

Thomas McFate, PhD is the Senior Educational Specialist for the Office of Faculty Development at the Uniformed Services University of the Health Sciences.

K. Christopher McMains, MD is an Otolaryngologist and Professor of Surgery at the Uniformed Services University of the Health Sciences. He is the Chair of the Faculty Development Subcommittee at the San Antonio Uniformed Services Health Education Consortium. His clinical work is at the South Texas Veterans Health Care System.

Rechell G. Rodriguez, MD is an Associate Professor of Medicine at the Uniformed Services University of the Health Sciences who has been a clinician educator for 20 years and has been involved in faculty development since 2010 after attending the Stanford Faculty Development Program. She helped expand faculty development in the Military Health System and is now providing faculty development for the VA San Diego Healthcare System.

Joshua D. Hartzell, MD, MS-HPEd is a Professor of Medicine at the Uniformed Services University of the Health Sciences. He currently serves as the National Capital Consortium Internal Medicine Residency Program Director at Walter Reed National Military Medical Center. He has an interest in faculty and leadership development. Twitter -@joshuadhartzell, ORCID: https://orcid.org/0000-0001-7135-1287
