# Supplementary material for: The Ripple Effect: A Train-the-Trainer Model to Exponentially Increase Organizational Faculty Development
Source: MedEdPublish (2016). 2020 Aug 5;9:158. [Version 1] doi: 10.15694/mep.2020.000158.1 (PMC10702681; doi:10.15694/mep.2020.000158.1)
Supplement: Peer review history

# Reviewer report: Reviewer response for version 1

**DOI:** 10.21956/mep.20078.r30894

**Published:** 2020-08-16

**Authors:** Helena Filipe

**Affiliations:** 1 Hospital of the Armed Forces/PL-EMGFA

This review has been migrated. The reviewer awarded 4 stars out of 5 The abstract overviews the Authors’ journey in designing, implementing and evaluating a faculty development program based on the Kolb’s cycle and “deliberate practice as a framework”. A brief mention of the TRAIN framework in the abstract would consistently link with the judicious explanation in the long discussion section and how this investigation methodology suits its structure. Throughout the manuscript we can feel the feeling of excitement, commitment and willingness of Authors to create safe collective and fulfilling learning spaces for "faculty trainers".The introduction builds well the case for the investigation ahead described and is appropriately evidence informed. Authors emphasise the need to provide evidence-based learning opportunities to all healthcare educators to meet increasing expectations and requirements from accrediting bodies. This work adds to the previous nationwide faculty development programs by expanding beyond a scalable central live experience to provide the opportunity to build a community of learning. The methodology is described to the point of replication and is evidence informed. A note of remark for the wonderful collection of curated bibliography the Authors share with the reader. The table shows well the topics approached during both learning programs. Probably a flowchart could be helpful to illustrate and visualize the overall research process used. In the results section a diagram could also be helpful.The discussion session could (perhaps) be summarised. A diagram combining the TRAIN framework components with the elements of this research as reasoned by the Authors would help the reader visualising why and how the methodology pertains this model. The TRAIN framework provides theoretical ground to justify the sustainability of this program in the future, ensuring its continuity, providing a longitudinal learning opportunity and nurturing a community of practice. In the conclusions section, Authors emphasize that their research is based on a deliberate practice framework and Kolb Cycle. Would like to see in the Take Home messages some allusion to the community of practice mentioned at the beginning.In summary the article focuses a relevant topic, overall reads well, is well organised and adds to previous science. Some suggestions are provided above. In a next paper, it will be good to see all cohorts sharing exactly the same instruction modules, community of practice follow up and feedback collected from the participants in programs conducted by the Faculty trainers of this faculty program.

Reviewer Expertise:

NA

No decision status is available

# Reviewer report: Reviewer response for version 1

**DOI:** 10.21956/mep.20078.r30897

**Published:** 2020-08-08

**Authors:** Megan Anakin

**Affiliations:** 1 University of Otago

This review has been migrated. The reviewer awarded 2 stars out of 5 The title of this article caught my attention. I am a faculty developer and I was curious about how a train-the-trainer model might be able to exponentially increase organizational faculty development.To strengthen the claims made in the opening paragraph of the introduction, please consider supporting the claims made about the increasing need for faculty development and skills needed by all educators with references to the literature. To enhance the introduction for the reader, the authors may wish to discuss the literature about the train-the-trainer programme before introducing the new methods’ context and aim. To help the readers understand the results that are reported, the authors may wish to consider describing the evaluation instruments, and the data collection and analysis procedures in the methods section. In the discussion section, the authors state, ‘Our train-the-trainer program has been an effective model to develop a community of national faculty developers’, however, missing from the earlier sections of this article is a description of the features that will be examined or measured to determine the effectiveness of the train-the-trainer programme. Please consider describing how the effectiveness of the programme was measured and specify the methods used. Please consider spelling out and defining the term SFDP is used in the second sentence of the discussion section to help the reader understand how it was used as a model for the train-the-trainer programme. Please consider introducing this term earlier in the article so the reader is familiar with it when it is used to make comparative comments in the discussion section. Please consider substantiating the claim in the discussion that ‘The number of workshops taught, and faculty attending have been significantly higher’ with statistical results of data analysis presented in the results section. Please consider presenting the evaluation questions used in the methods section so that reader can better understand how the evaluation results presented in the results section support the claim ‘The survey results of faculty performance imply that even with only a week of dedicated training our faculty trainers were able to deliver the material effectively’ in the discussion section. Please consider explaining the link between this claim and the suggestion ‘that others could develop a similar more condensed train-the-trainer program for faculty development at their institution’. Alignment between the train-the-trainer programme and the TRAIN framework is explained in the discussion section, however, to enhance the relevance of this explanation for the reader, the authors may wish to make more explicit links to the evaluation results of their study. Please consider introducing the TRAIN model in the introduction section so the reader is familiar with this model before the discussion section. Please consider discussing several methodological challenges and limitations of your evaluating your new method along with those identified for others who might be planning to implement a similar programme. Please consider revising the first sentence of the conclusion to better reflect the results of the study. I would be very happy to review a revised version of this article.

Reviewer Expertise:

NA

No decision status is available

# Reviewer report: Reviewer response for version 1

**DOI:** 10.21956/mep.20078.r30896

**Published:** 2020-08-08

**Authors:** Subha Ramani

**Affiliations:** 1 Harvard Medical School, Brigham and Women's Hospital

This review has been migrated. The reviewer awarded 3 stars out of 5 I enjoyed reading this article and recognize how challenging it would be to train large cohorts of busy clinical teachers and engage in faculty development initiatives with a scope such as this. Faculty development of clinical teachers at a multitude of sites, each with their own unique context and challenges, is quite a large undertaking. I agree that developing faculty trainers who can then develop other faculty is a good model to achieve this.The program required significant amount of in-person time and I applaud the authors for completing the program. I wonder, however, if this time will be consistently available in the future and recommend that the authors start thinking about a blended model of delivery. As they state, this program needs resources and support from leadership to be sustainable long-term.I am quite familiar with the Stanford model, having trained in the program myself, and the leaders of that program have developed hundreds of faculty trainers over the course of 20+ years. Their model was brilliant, but required time to implement well.The theoretical frameworks employed by the authors are appropriate for a faculty development experience designed for clinical educators.The authors have also described well the key aspects of faculty development programs in their discussion section- talent, resources, alignment, nurturing etc.I do have a few constructive comments:- I do not see a clear needs assessment plan which guided development of the program content. - I agree that the TRAIN model is thrust into the discussion rather abruptly without a prior explanation. I always wonder if too many models, theories or frameworks may confuse readers. We have the deliberate practice and Kolb's experiential model already anchoring the design, do we need another?- Program evaluation is the weakest part of this initiative. A very brief nod to participant satisfaction is all that I see. Since nurturing seemed to be an important aspect of the program, some self-reports of behavior change might be useful, thus targeting higher levels of Kirkpatrick pyramid of outcomes. - Finally, I would like some recommendations from the authors to other educators with less resources on how they may adapt a program such as this to their own local context.- Though deliberate practice and Kolb's model are mentioned, the authors could make the link between principles and their application more clear and obvious- especially for those who are less familiar with the details of these models. What steps of the program mirrored what principles, for example.All in all, this article would be useful for all those engaged in faculty development of clinical educators. But readers would need to distill what aspects of this model would be feasible and applicable to their own context.

Reviewer Expertise:

NA

No decision status is available

# Reviewer report: Reviewer response for version 1

**DOI:** 10.21956/mep.20078.r30895

**Published:** 2020-08-05

**Authors:** Felix Silwimba

**Affiliations:** 1 University of Lusaka

This review has been migrated. The reviewer awarded 5 stars out of 5 very important approach to effort at improving the quality of medical educators.it is an approach worth trying in low income countries .

Reviewer Expertise:

NA

No decision status is available
